# Lymphocytosis and Mycobacteriosis in a 15‐Year‐Old Mixed‐Breed Dog

**DOI:** 10.1155/crve/5288911

**Published:** 2026-02-26

**Authors:** Bridgette J. Murphy, Ashley A. Smith, Erin M. Kilbane, Francisco O. Conrado

**Affiliations:** ^1^ Department of Pathobiology, Auburn University College of Veterinary Medicine, Auburn, Alabama, USA, auburn.edu; ^2^ Department of Clinical Sciences, Auburn University College of Veterinary Medicine, Auburn, Alabama, USA, auburn.edu; ^3^ Department of Comparative Pathobiology, Cummings School of Veterinary Medicine at Tufts University, North Grafton, Massachusetts, USA

## Abstract

A 15‐year‐old mixed‐breed dog with T‐cell lymphoid neoplasia with persistent CD8^+^ lymphocytosis and peripheral lymphadenopathy despite chemotherapy was referred to the Auburn University Veterinary Teaching Hospital Oncology service approximately 7 months after diagnosis. Lymph node aspirates were performed and assessed by a board‐certified clinical pathologist, which revealed granulomatous inflammation and intracellular, negative staining bacilli. Bacilli were confirmed *Mycobacterium avium* and other species within the *M. avium* complex by polymerase chain reaction (PCR) testing. Chemotherapy was ceased and antimicrobial therapy was initiated; however, the lymphocytosis continued to progress, and the dog began to decline, leading to euthanasia around 2 months after referral. Although T‐cell lymphoid neoplasia and mycobacteriosis are rare comorbidities, this case highlights the importance of thorough cytological assessment of lymph node aspirates as part of a complete workup to rule out differential causes of lymphadenopathy. This may have allowed for earlier diagnosis and treatment of mycobacteriosis.

## 1. Introduction

Lymphoid neoplasia is a heterogeneous group of diseases that most commonly arise in lymphoid tissues (i.e., lymph nodes, spleen, and bone marrow) [1, 2]. Lymphoma and leukemia are distinguished based on the tissue distribution of neoplastic cells; however, the presence of circulating neoplastic cells in peripheral blood may be indicative of lymphoma with bone marrow involvement (i.e., stage V disease) or primary lymphoid leukemia [1, 2]. Peripheral lymphadenopathy can be seen in both, and treatment usually involves various chemotherapeutic protocols [1].

Mycobacteriosis is a differential diagnosis for lymphadenopathy. Mycobacteria are grouped into the *Mycobacterium tuberculosis* complex (MTBC), the *Mycobacterium avium* complex (MAC), the lepromatous mycobacteria, and nontuberculous mycobacteria (NTM) other than *Mycobacterium avium* [3]. MAC organisms are found in soil, dust, and aquatic environments [3]. They are slow‐growing organisms that rarely cause cutaneous and disseminated granulomatous disease in dogs, and infection is most often associated with immunocompromised individuals [4].

The aim of this case report is to demonstrate the importance of performing a thorough diagnostic workup to rule out differential causes of lymphadenopathy. The development of peripheral lymphadenopathy in this dog was attributed to possible microbiological disease, for which doxycycline was prescribed; however, no further diagnostic testing was carried out. Upon referral to Auburn University Veterinary Teaching Hospital (AUVTH) and assessment of lymph node aspirate cytology by a board‐certified clinical pathologist, intracellular, acid‐fast bacilli were seen, which were identified as *Mycobacterium avium* and others within the *M. avium* complex by polymerase chain reaction (PCR). If mycobacteriosis had been diagnosed earlier, appropriate antimicrobial therapy may have been instituted promptly, potentially improving disease control, reducing reliance on chemotherapeutics, and decreasing treatment‐related toxicity.

## 2. Case Presentation

A 15‐year‐old spayed female Labrador mix was referred to the AUVTH Oncology Service in June 2024 for a second opinion on T‐cell lymphoid neoplasia. Initial diagnosis occurred in November 2023, with an incidental finding of lymphocytosis (21 x 10^3^/*μ*L; reference interval [RI], 0.69–4.50) characterized by an expansion of intermediate‐sized lymphocytes. Flow cytometric analysis was performed on peripheral blood [5] using the Sony ID7000 spectral flow cytometer (Sony Biotechnology Inc.), and results were consistent with a CD8^+^ lymphocytosis (Figure 1a). PCR for antigen receptor rearrangement (PARR) was not performed on the blood to confirm clonality. There were no antibodies to *Ehrlichia* spp. (*Ehrlichia canis*, *Ehrlichia ewingii, and Ehrlichia chaffeensis*) detected using the Accuplex test (Antech). Chlorambucil and prednisone were initiated on November 25, 2023. The lymphocytosis improved after 1 month (9 x 10^3^/*μ*L), prompting dose reduction of chlorambucil and discontinuation of prednisone. Peripheral lymphadenopathy was first noted in December 2023 and attributed to possible infection, for which doxycycline was prescribed. No fine‐needle aspirates (FNA) of the enlarged lymph nodes were performed at this time. Chlorambucil dose adjustments were made over subsequent months due to mild progression of the lymphocytosis (14.8 x 10^3^/*μ*L). By April 2024, lymphocytosis progression (22 x 10^3^/*μ*L) and persistence of peripheral lymphadenopathy prompted transition to the CHOP (cyclophosphamide, doxorubicin, vincristine, and prednisone) protocol. FNA of a popliteal lymph node were performed and assessed by cytologic evaluation by the primary clinician, which were noted to contain a population of small lymphocytes. By the end of May 2024, the lymphocytosis had resolved (3.9 x 10/*μ*L). Referral was pursued due to static lymphadenopathy and severe chemotherapy‐associated gastrointestinal adverse events, which included abdominal distension, vomiting, and diarrhea. The dog lived mostly indoors, was fed a commercial diet, had one healthy canine housemate, and had no contact with birds, wildlife, or bovines.

Figure 1Flow cytometry of peripheral blood performed in November 2023 (a) and July 2024 (b), showing that the majority of lymphocytes are CD4^−^, CD5^+^, and CD8^+^ T‐lymphocytes in both samples.

**Figure:**
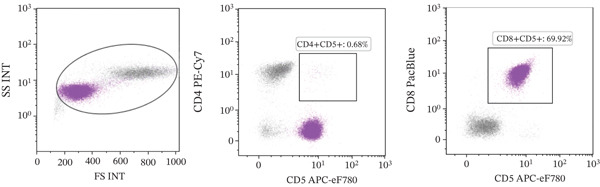
(a)

**Figure:**
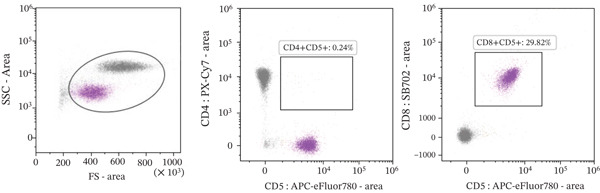
(b)

### 2.1. Clinical and Clinicopathologic Findings

Physical examination at the AUVTH Oncology Service found mild peripheral lymphadenopathy of the mandibular, superficial cervical, and right popliteal lymph nodes (17–22 mm), hepatomegaly, mild diffuse muscle atrophy, and several subcutaneous masses cytologically identified as lipomas. A CBC (Advia 2120i, Siemens Healthcare) and serum biochemistry panel (Cobas c 311, Roche Diagnostics) revealed a lymphocyte count within reference interval (WRI), an expansion of intermediate‐sized lymphocytes (Figure 2), a mild normochromic, macrocytic, nonregenerative anemia (PCV, 34.7%; RI, 38.7–59.2; absolute reticulocyte count, 43.4 x 10^3^/*μ*L; RI, 0.0–60.0), and moderately elevated alkaline phosphatase activity (409 U/L; RI, 14–152). FNA of the enlarged right superficial cervical and right popliteal lymph nodes were obtained and submitted for review by a clinical pathologist (Figure 3). Three‐view thoracic radiographs were radiographically unremarkable with no evidence of metastasis or lymphadenopathy. Abdominal ultrasound revealed a subjectively enlarged liver with rounded margins and multiple ill‐defined hyperechoic and hypoechoic nodules measuring less than 6 mm, mildly heterogeneous medial iliac lymph nodes, and multiple cystic abdominal lymph nodes. The liver was interpreted as a benign vacuolar/nonspecific hepatopathy, and the lymph nodes were interpreted as reactive per the board‐certified radiologist, but FNA of these sites was not performed. Peripheral blood was submitted for flow cytometric and PARR analyses. Flow cytometry identified a homogeneous expansion of CD8^+^ T cells most consistent with CD8^+^ T‐cell lymphoid neoplasia (Figure 3b). The PARR assay identified a clonally rearranged T‐cell receptor gene [6], strengthening the diagnosis of neoplasia (Figure 4).

Figure 2Photomicrographs of peripheral blood at (a) 50x oil magnification; bar = 20 *μ*m and (b) 100x oil magnification; bar = 10 *μ*m. There is an expansion of intermediate‐sized lymphocytes (approximately half the lymphoid population). Lymphocytes are round to ovoid, with discrete cytoplasmic borders, a high nucleocytoplasmic ratio, and a small to moderate amount of pale to medium basophilic cytoplasm. The nucleus is paracentral to eccentric and measures approximately 1.5–2x the size of an erythrocyte. Chromatin is smooth to clumped and a rare, indistinct nucleolus is noted. Wright–Giemsa.

**Figure:**
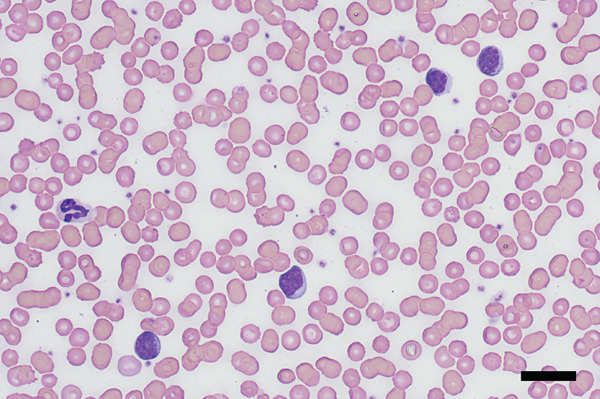
(a)

**Figure:**
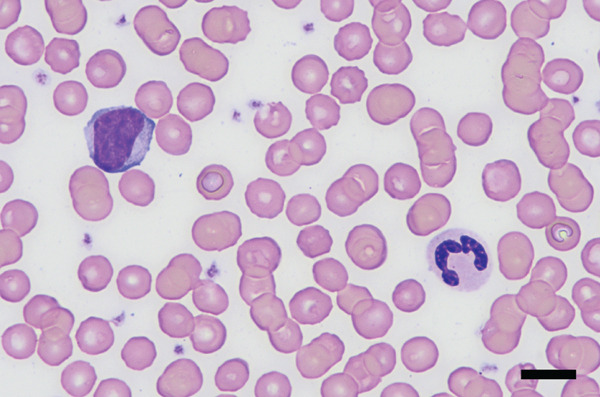
(b)

Figure 3Photomicrographs of fine needle aspirates from the lymph nodes showing (a) aggregates of epithelioid macrophages, variably degenerate neutrophils, occasional plasma cells, and a heterogeneous lymphoid population (reactive lymphoid hyperplasia). Wright–Giemsa stain; bar = 20 *μ*m. (b) negative staining bacilli within macrophage cytoplasm and free in the lightly basophilic background (black arrows). Wright–Giemsa stain; bar = 10 *μ*m.

**Figure:**
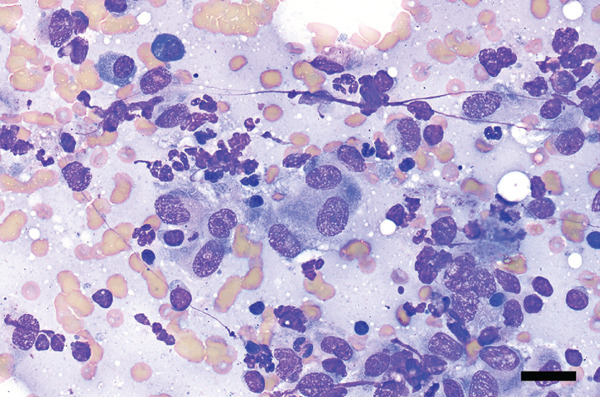
(a)

**Figure:**
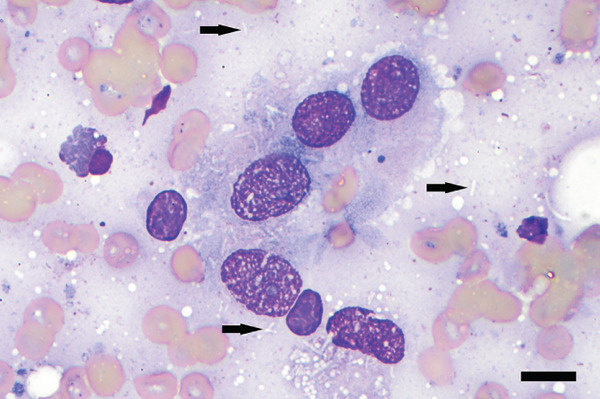
(b)

Figure 4Polymerase chain reaction (PCR) for antigen receptor rearrangement assay of the T‐lymphocyte receptor gamma gene is shown. (a) Polyclonal T‐lymphocyte receptors in peripheral blood. (b) Clonal T‐lymphocyte receptors in the dog.

**Figure:**
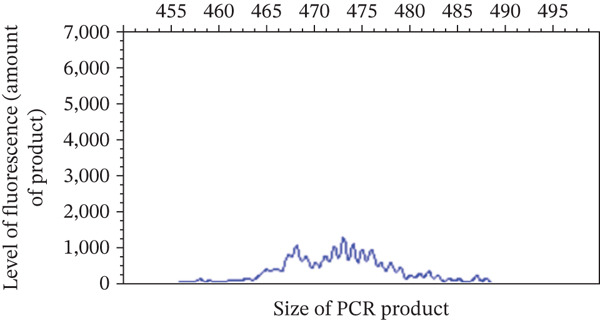
(a)

**Figure:**
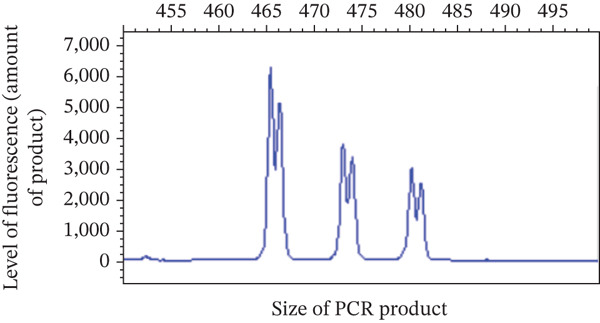
(b)

### 2.2. Cytologic and Microbiological Findings

**Figure 5:**
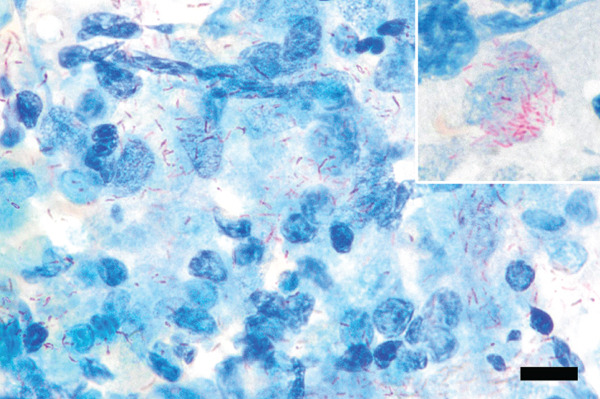
Photomicrographs of acid‐fast stain of lymph node aspirates. Free acid‐fast bacilli are seen within the background among cellular material. Ziehl–Neelsen (ZN) acid‐fast stain; bar = 20 *μ*m. Inset—A macrophage with intracellular acid‐fast bacilli within the cytoplasm. Ziehl–Neelsen (ZN) acid‐fast stain; x100 oil objective.

Microscopic examination of Wright–Giemsa–stained slides from aspirates of the right superficial cervical lymph node and the right popliteal lymph node (Figure 3) revealed many heavily vacuolated macrophages that occasionally contained phagocytized, negatively stained bacilli. Macrophages were often seen in epithelioid aggregates, and occasional negative‐staining free bacilli were noted in the lightly basophilic background. The lymphocyte population was predominantly composed of small lymphocytes, with fewer intermediate‐ and large‐sized lymphocytes. There were a moderate number of neutrophils that were variably degenerate (karyolytic). No overtly neoplastic cells were seen. The cytological findings in both lymph nodes were interpreted as pyogranulomatous inflammation with intracellular negative‐staining organisms most consistent with *Mycobacterium* spp. Additionally, the heterogeneous lymphoid population in the right popliteal lymph node was interpreted as reactive lymphoid hyperplasia. Ziehl–Neelsen acid‐fast stain of the lymph node aspirates showed few acid‐fast organisms (Figure 5). Additional diagnostics were recommended to confirm the suspicion of mycobacteriosis, such as PCR and surgical biopsy with immunohistochemistry.

Lymph node fluid obtained by fine needle aspiration was submitted to Washington Animal Disease Diagnostic Laboratory (WADDL) for culture and PCR testing. Aspirated fluid was cultured using Middlebrook 7H11 agar and Middlebrook 7H9 broth (Hardy Diagnostics, Santa Maria, CA). After inoculation, both agar and broth were incubated at 35°C in 5% CO_2_. At 72 h of incubation, pinpoint growth was observed on the Middlebrook 7H11 agar. At 7 days the subculture showed pure bacterial growth with smooth, flat, transparent colonies. Ziehl–Neelsen acid‐fast stain was performed, and acid‐fast positive bacilli were observed. A portion of the 16S‐23S ribosomal internal transcribed spacer region was amplified by PCR using universal *Mycobacterium* primers [7]. The sequence most closely matched that of *M. avium* and others within the *M. avium* complex (100% sequence identity) when compared with sequences in GenBank. These primers were unable to differentiate between subspecies in the MAC.

Following the diagnosis of CD8^+^ T‐cell lymphoid neoplasia with concurrent mycobacteriosis, the dog was started on clarithromycin (11 mg/kg, PO, q 12 h) and enrofloxacin (9 mg/kg, PO, q 24 h) with the intent to administer these antimicrobials for 6–9 months [8]. Rifampin was not started due to concern for hepatotoxicity with chronic administration and the unlikely ability to clear the infection in an immunocompromised host. Further chemotherapy including corticosteroids was not pursued. The zoonotic risk was discussed with the owner, quarantine instructions were provided, and they were advised to consult with their primary physician. The lymphocytosis continued to progress (Table 1), and the dog began to decline, leading to euthanasia around 2 months after referral.

**Table 1:** A summary of complete blood count (CBC) results showing the progression of lymphocytosis after referral to AUVTH.

Test	June 12, 2024 (AU)^a^	June 28, 2024 (RDVM)^b^	July 5, 2024 (RDVM)^b^	August 3, 2024 (RDVM)^b^
Hematocrit (%)	**34.7** (38.7–59.2)	39 (36–60)	40 (36–60)	**33** (36–60)
Platelets (x10^3^/*μ*L)	414 (152–518)	WRI (170–400)	**565** (170–400)	371 (170–400)
White Cell Count (x10^3^/*μ*L)	15.94 (5.09–17.40)	**45.30** (4.0–15.50)	**56.30** (4.0–15.50)	**383.40** (4.0–15.50)
Neutrophils (x10^3^/*μ*L)	8.12 (2.6–10.40)	**13.13** (2.06–10.40)	6.20 (2.06–10.40)	3.83 (2.06–10.40)
Lymphocytes (x10^3^/*μ*L)	6.37 (0.39–6.73)	**30.80** (0.69–4.50)	**40.50** (0.69–4.50)	**379.56** (0.69–4.50)

*Note:* Bolded numbers represent results outside of the provided reference interval.

Abbreviations: AU, Auburn University; RDVM, primary clinician, WRI, within reference interval.

^a^ ^a^Advia 2120i analyzer, Siemens Healthcare. Reference intervals: Auburn University Clinical Pathology Laboratory.

^b^ ^b^IDEXX Procyte Dx Veterinary CBC Hematology Analyzer reference intervals.

## 3. Discussion

The diagnosis of T‐cell lymphoid neoplasia was further complicated by mycobacteriosis in this dog. The distinction between lymphoma and leukemia is based on the extent of disease. In this case, lymphoid neoplasia appeared confined to the blood; however, aspirates of the liver, spleen, and bone marrow were not performed to further characterize the disease.

Williams et al. [9] reported a significantly shorter median survival time (MST) in dogs presenting with a CD8^+^ lymphocytosis of >30 x 10^3^/*μ*L (MST = 131 days) compared with those presenting with <30 x 10^3^/*μ*L (MST = 1098 days). At the time of diagnosis, this dog′s lymphocytosis was reportedly around 21 x 10^3^/*μ*L and was an incidental finding. Although lower than the proposed threshold, this dog′s survival was significantly shorter. Treatment with chlorambucil and prednisone was initially pursued and resulted in improvement of the lymphocytosis. The discontinuation of corticosteroids after 1 month may have contributed to the short duration of disease control. Alternatively, the initial mild fluctuations in lymphocyte count (9–22 x 10^3^/*μ*L) may be attributed to the natural disease course of an indolent leukemia. The dog′s clinical disease course accelerated after chemotherapy was discontinued. The impact of concurrent mycobacteriosis on progression of lymphoid neoplasia is unknown.

MAC is the most common of the NTM and is composed of slow‐growing NTM species that are ubiquitous in the environment for example, soil and water [10]. There are many species within the MAC, and they are known to cause skin, respiratory, gastrointestinal, and disseminated infections in immunocompromised hosts [4, 10, 11]. Dissemination of mycobacteriosis results in disease progression, and treatment attempts are typically unsuccessful [10]. Lymphadenopathy was noted ultrasonographically in the abdominal lymph nodes, potentially due to disseminated mycobacteriosis; however, this was not confirmed with further testing. Once mycobacteriosis was diagnosed in this dog, chemotherapy was ceased, and antimicrobial therapy was initiated. The lymphocytosis progressed quickly (Table 1), and rapid clinical decline resulted in euthanasia (~270 days after diagnosis). The combination of T‐cell lymphoid neoplasia and the added complication of potentially disseminated MAC mycobacteriosis resulted in this dog′s relatively short survival time. It is not known whether mycobacteriosis was present prior to lymphoid neoplasia, and immunosuppression led to clinical disease, or if immunosuppression led to susceptibility to mycobacteriosis.

This case highlights the importance of performing thorough cytological examination of lymph node aspirates and considering infectious differential causes for lymphadenopathy. Earlier diagnosis of mycobacteriosis in this dog would have allowed prompt treatment of infection, which may have improved control of mycobacteriosis, reduced reliance on chemotherapeutic agents, and decreased the severe treatment‐related gastrointestinal side effects. Importantly, the dog posed a zoonotic risk for *Mycobacterium* spp. infection while the infection went undetected. This complicated handling of the dog within multiple hospitals, including exposure to immunosuppressed chemotherapy patients. Although interspecies transmission of NTM has not been definitively described, retrospective contact tracing is challenging due to the complex epidemiology (i.e., environmental ubiquity, many routes of transmission, broad host range, variable clinical presentation, and long incubation period) [10], therefore, zoonotic risk should be taken seriously and potential contacts should be advised to seek advice from their primary care physician [10].

## Funding

No funding was received for this manuscript.

## Conflicts of Interest

The authors declare no conflicts of interest.

## Data Availability

The data that support the findings of this study are available from the corresponding author upon reasonable request.
